# Chronoprevention in hospital falls of older people: protocol for a mixed-method study

**DOI:** 10.1186/s12912-021-00618-y

**Published:** 2021-06-06

**Authors:** Pablo Jesús López-Soto, Juan de la Cruz López-Carrasco, Fabio Fabbian, Rosa María Miñarro-Del Moral, Rocío Segura-Ruiz, Pedro Hidalgo-Lopezosa, Roberto Manfredini, María Aurora Rodríguez-Borrego

**Affiliations:** 1 Department of Nursing, Maimonides Biomedical Research Institute of Cordoba (IMIBIC), Avda. Menéndez Pidal s/n, 14004 Córdoba, Spain; 2 Área de Enfermería, Faculty of Medicine and Nursing, University of Cordoba, Avda. Menéndez Pidal s/n, 14071 Córdoba, Spain; 3 Hospital Universitario Reina Sofía de Córdoba, Avda. Menéndez Pidal s/n, 14004 Córdoba, Spain; 4 Faculty of Medicine, Pharmacy and Prevention, University of Ferrara, Via L. Ariosto 35, 44121 Ferrara, Italy; 5 Center for Studies on Gender Medicine, University of Ferrara, via Fossato di Mortara 64/b, 44121 Ferrara, Italy

**Keywords:** Accidental falls, Older people, Nursing care, Nursing, Temporal patterns, Prevention, Circadian rhythms

## Abstract

**Background:**

Accidental falls in hospitals are serious events concerning the safety of the patients. Recent studies demonstrated that the time of falls is a key factor to be considered in prevention. It has been shown that the time of day, the day of the week and the month of the year impact on the occurrence of falls. The aim of the study is to determine the effect of the application of a programme of preventive measures based on the temporal patterns of the risk factors on the hospital fall occurrence.

**Methods:**

A mixed-method research design. The following three phases will be carried out: 1) Longitudinal prospective study in two parts: (a) audits and seminars of healthcare professionals focused on an effective and efficient hospital falls register. Multi-Component and Single Cosinor analyses will be performed to obtain the temporal patterns of hospital falls and their related variables and (b) implementation of a based-temporal patterns, multidimensional prevention programme. 2) Retrospective study of falls registered in institutional databases. 3) Qualitative study based on focus groups (physicians, nurses and nursing assistants). The study protocol was approved in 2018.

**Discussion:**

With regard to the safety of patients, hospital falls are serious events. Recent studies have demonstrated that the time of falls is a key factor to be considered in prevention. It has been shown that the time of day, the day of the week and the month of the year impact on the occurrence of falls. It is imperative to study temporal patterns of hospital falls to effectively and comprehensively define the aetiology of falls and, therefore, design preventive strategies. A reduction of the number of in-hospital falls and related injuries is expected, as well as an improvement in the quality of life of patients. Considering temporal patterns and levels of mood and sleep of healthcare professionals will achieve an improvement in patient safety.

**Trial registration:**

Retrospectively registered in ClinicalTrials.gov ID: NCT04367298.

**Supplementary Information:**

The online version contains supplementary material available at 10.1186/s12912-021-00618-y.

## Background

The proportion of older people in the general population has increased during the last few years. Consequently, an important challenge of the public health system is to preserve the autonomy and independence of this group. One of the greatest causes of loss of autonomy and independence are accidental falls and related injuries. Epidemiology shows that accidental falls are not only a main and common problem among older people all around the world but also a big issue in terms of social and economic repercussions for the victims and their families. [1]

Hospital falls imply a greater challenge for healthcare organizations due to their effect on the patient’s quality of life (due partly to the loss of autonomy and independence). In addition, they increase morbidity and length of stay in hospitals, leading to an important rise of the sanitary outlay [2, 3].

Falls in older people are due to intrinsic and extrinsic factors [4]. Even though many studies have been conducted to identify the causality of falls, the time of falls is often not considered [5]. Evidence suggests temporal patterns in the occurrence of hospital falls [6]. Research in temporal patterns in the manifestation and intensity of symptoms and risk of life-threatening and life-ending events (chronopathology and chronorisk) is essential to develop longitudinal (chrono)preventive and (chrono)therapeutic interventions and strategies that take into account the aforementioned patterns [7, 8]. Therefore, this study aims to implement longitudinal preventive counter-measures based on temporal patterns of incident events and relative risk factors for falls and related injuries (*chronoprevention*).

The risk of fall is directly correlated to ageing, so the number of accidental falls will proportionally grow with the ageing of general population [4, 9]. Recent studies show that 30 % of people living among their communities suffer one fall per year [10], rising to 40 % among hospitalized people [11]. Regarding hospitalized people, fall rates show a great variability, between 2 and 17 falls per 1,000 occupied beds [12, 13].

Accidental falls are the result of a complex interaction of multiple risk factors [4]. Lately, numerous epidemiological studies about accidental falls have identified intrinsic risk factors (biological and behavioural), such as age, chronic medical conditions, drug adverse effects and wrong lifestyles, and external risk factors (socioeconomic and environmental, a large part of which are modifiable), for instance, housing quality and design, house surface and illumination [14–16]. According to Cameron [17], an effective intervention, even when only assessing one associated risk factor, could reduce both the number of events and the associated morbidity [18].

### Temporal patterns in hospital falls and relative risks of fall

Although important efforts have been made to identify possible risk factors for falls, the time of falls has often been ignored. With regard to the occurrence of accidental falls, it is important to know not only the time of day and the shift work of the healthcare professionals (especially nurses, who are responsible for the data collection) but also the relative risk of intrinsic and external factors (*chronoepidemiology*), as well as circadian rhythms related to the clinical care of patients and healthcare workers [19, 20]. Recent studies of our research group [5, 20, 21] observed that the time of the day, the day of the week and the month of the year have impacts on the fall occurrence. Concretely, a retrospective study [20], carried out in five different non-teaching public Italian hospitals after the development of a programme to prevent falls, identified 24-hour and seasonal patterns for accidental falls in hospitals among a population older than 65 years old and showed crucial information about the relationships between the circumstances of falls (cause, mode and place) and times of falls. On the other hand, the extension of preventive measures according to the obtained temporal patterns (*chronoprevention*) affected the reduction of the number of falls in hospitals, although, due to the retrospective nature of the study and the association with other institutional measures not included in the study, a possible direct effect was not established.

In addition to the research carried out in our group, several studies have been focused on the topic, which is an important indicator of the increasing importance of the research field among the scientific community [22, 23]. Both studies confirmed the finding of a morning peak in hospital falls strictly related to bathing activities. In fact, as consequence of the results published by Magota [23], a letter to the editor was published in order to promote more comprehensive research about accidental falls focused on temporal patterns [24].

It is important to emphasize that some patient characteristics, such as number and type of medical conditions, have some influence on the time of falls in hospitals [7, 20]. The type of drug has some influence on the temporal pattern of falls [8]. Nevertheless, it is important to understand whether the administration time of the drug has an influence on the fall occurrence and, therefore, to reduce its incidence. This is why understanding the temporal differences in the administration of hypertension drugs led to a reduction of different clinical conditions (cardiovascular diseases, diabetes mellitus or kidney failure) [25].

### In-hospital falls and registration culture

Documenting and collecting data about the causes and circumstances of accidental falls is fundamental to produce an optimal preventive programme. Another study [26] proved not only the lack of data collecting about accidental falls in Spanish hospitals and community settings but also the lack of registration of the fall. This is probably because most of the falls do not require medical attention or they have not been witnessed. Moreover, healthcare personnel perceive that registration of the fall is a nurse’s task, or they totally ignore that something should be done. Another key factor is the excess load of work of the healthcare staff, and therefore, the lack of time to properly produce an appropriate register. Hence, it is important to support, teach and educate healthcare personnel about proper fall documentation, including the circumstances. In this sense, the improvement of an intuitive, simple and uniform system would allow the register to be used successfully, leading to the possibility, a posteriori, of research and data collecting and analysis about temporal patterns, which could create a more appropriate perspective of risks and fall-related injuries in this population [27].

### Chronoprevention: a nursing intervention

To our knowledge, there are no epidemiological studies proposed with a chronobiological approach to demonstrate the influence of preventive counter-measures adjusted to temporal patterns on the incidence of accidental falls [28]. Therefore, it is necessary to develop prospective studies in order to confirm temporal patterns in hospital falls and to fully understand intrinsic and extrinsic risk factors.

Lastly, the temporal patterns of the professional team, including the number of qualified workers, the fulfilment of patient caring tasks and the characteristics of their work schedule, form another important factor to be considered during the examination of time patterns in accidental falls in hospitals.

Generally, healthcare worker teams are reduced in number during night shifts, weekends and holidays [21]. Some programmed tasks and activities of patient care have an influence on the progress of the fall, such as bed sheets changing, bathing, feeding or transferring [20].

Besides, the roles of fatigue/sleepiness, attention and cognitive functionality in healthcare professionals, just like disruptor agents of circadian time structure (i.e. shift work, night shift, etc.), could affect the vigilance and attention to falls during the day and night. Therefore, it is necessary to evaluate the aforementioned variables in order to define effectively and efficiently the aetiology of falls and, based on emerging temporal patterns, to implement preventive measures in order to reduce the occurrence of hospital falls.

## Methods/design

### Aims

The main aim of this study will measure the effect of the implementation of a falls prevention programme based on temporal patterns and relative risks factors.

Secondary aims will be: (i) to identify temporal patterns in the incidence of hospital falls and intrinsic and external risks factors, pre- and post-intervention; (ii) to establish differences in temporal patterns according to the hospital setting (teaching or non-teaching) and the different types of wards (medical, surgical or mixed), pre- and post-intervention; (iii) to study the role of circadian time in drug administration for older people suffering from hospital falls, pre- and post-intervention; (iv) to identify the level of fatigue/sleepiness, attention and cognitive functionality of healthcare professionals intervening during hospital falls, pre- and post-intervention; and (v) to develop a uniform, simple and intuitive method to record hospital falls in order to improve the effectiveness of the registration of falls and to assess the implementation pre- and post-intervention.

### Design

A mixed-method research design will be carried out. Three different study designs will be used:

***Phase 1.*** Prospective longitudinal study in two steps:

- A first step of 18 months, in which a first intervention of audits and seminars in healthcare professionals will be carried out, focused on an effective and efficient registry of hospital falls in older people. This first phase is crucial to identify temporal patterns of falls in relation to specific intrinsic and extrinsic risk factors among hospitalized older people, as well as the levels of fatigue/sleepiness, attention and cognitive functionality of the healthcare professionals who attend to an in-hospital fall.
- A second step of application of a multidimensional prevention programme for 15 months focused on organizational, educational and behavioural elements applied to admitted older people and healthcare professionals.

***Phase 2.*** Retrospective study of the fall records found in the institutional databases of the study centres in the period between January 2018 and September 2020. This period covers the first (January 2018–May 2019) and second phases (June 2019–September 2020) of the project, as previously indicated.

***Phase 3.*** Descriptive exploratory study with a qualitative approach, focus groups made up of healthcare professionals: doctors, nurses and nursing assistants who provide and have provided their professional services in the field of study, in the periods in which the longitudinal and retrospective studies have been carried out.

### Participants and setting

***Phase 1.*** The study subjects will be all those patients over 65 years of age who suffer one/several fall(s) during their admission to the Reina Sofia University Hospital in Córdoba and in the three regional hospitals of the Province of Córdoba (Cabra, Montilla and Pozoblanco) from January 2018 to September 2020.

Specifically, for the study of the levels of fatigue/sleepiness, attention and cognitive functionality of the healthcare professionals, the reference nurses of the older person who has an in-hospital fall during the study period will also be included.

***Phase 2.*** Institutional records of falls occurring in patients over 65 years old. In order to contrast with the prospective design, the institutional records of the four hospitals will be collected during the same period (January 2018 to September 2020).

***Phase 3.*** Healthcare professionals (doctors, nurses and nursing assistants) will be interviewed.

### Inclusion and exclusion criteria

***Phase 1.*** Regarding patients, they should have been admitted in the aforementioned hospitals, should be over 65 years old and suffer an in-hospital fall. Furthermore, they should sign an informed consent accepting to participate in the study.

Healthcare professionals should have the following requirements: to sign an informed consent accepting to take part in the study and to be nurses in charge of the care of older people suffering from hospital falls, despite not witnessing the event.

Patients who suffer from hospital falls and healthcare professionals who take care of them will be included in the study continuously, which means after every single fall event.

In both cases (patients and healthcare professionals), the sampling will be total.

***Phase 2.*** Records of falls suffered in patients over 65 years old collected in different institutional databases (adverse event report, clinical record of the patient…) in the four hospitals. As in phase 1, the sampling will be total.

***Phase 3.*** Selection of healthcare professionals will be intentional and with the leverage of the collaboration with the centres involved. One person for each sector (doctors, nurses and nursing assistants). Inclusion criteria will be: (i) healthcare professional who has worked in the four hospitals during the time the longitudinal study is carried on; (ii) professionals of clinical units where hospital falls will be recorded during the longitudinal study; and (iii) professionals of clinical units in that are important to the risk of in-hospital falls, by pathologies, dependence, motor or mobility disorder, etc.

### Study variables

The main dependent variable will be the number of hospital falls. A hospital fall will be defined as “the consequence of a non-intentional and sudden movement towards the floor from a more elevated position” in hospital settings.

Other independent variables related with the patient who experiences a hospital fall include: (i) sociodemographic and economic data (age, gender, marital status and level of education); (ii) time (hour and minute, day and shift work) of fall; (iii) patient position (from the bed with or without bedrails, sitting, walking, and so on) and witness of the fall (roommate, family member, healthcare professional or no witness); (iv) use/no use of footwear and kind (open or closed); (v) cause of fall (instability (general instability and balance loss), dizziness (syncope, fainting and cardiovascular accident), drug adverse effects (diuretics, anti-hypertension and other drugs), accident (event with no identifiable cause), ambient-related (dropped objects, obstacles and wet floor), fragility (extreme fragility or weakness) or others); (vi) location of the fall (hospital room, bathroom, corridor, public area or others); (vii) fall-related injuries and severity (no wound/no consequences, minor wounds (grazes and minor contusions), afflicting wounds (contusions requiring stitches, fractures and skull trauma) or others (not afflicting wounds but still in need of minor medical intervention)); (viii) number and kind of coexisting clinical conditions; (ix) prescribed drugs and their time of administration; (x) chronotype measured by the Horne-Östberg Questionnaire (1976); (xi) quality of life in terms of body functionality, physical limitations, body pain, social role or function, mental health, emotional limitations, vitality, energy or fatigue and general perception about own health; and (xii) general health status in terms of somatic symptoms, anxiety, insomnia, social dysfunction and depressive symptoms.

In the case of healthcare professionals, independent variables will be: (i) sociodemographic and economic data (age, gender, marital status, level of education, work experience and time worked in the unit); (ii) kind of contract and work unit; (iii) sleep pattern (quality and quantity of hours); (iv) chronotype measured by the Horne-Östberg Questionnaire (1976); (v) quality of life in terms of body functionality, physical limitations, body pain, social role or function, mental health, emotional limitations, vitality, energy or fatigue and general perception about own health; (vi) general health status in terms of somatic symptoms, anxiety, insomnia, social dysfunction and depression; and (vii) fatigue/sleepiness, attention and cognitive functionality.

Pre- and post-intervention will be:

***Step 1.*** Before implementing preventive measures: development of audits and seminars in order to improve, secure and control the registration of falls in hospitals and related factors, as well as others study variables (quality of life, functional status, chronotype, fatigue/sleepiness, attention and cognitive functionality). These interventions will be carried out by research nurses who are working in hospitals.

***Step 2.*** Implementation of a preventive measures programme: adjusted to the identification of temporal patterns of falls and relative risk factors of falls. As measures will be agreed with the managers of hospital centres, the interventions will be reflected in the official document of general objectives of the hospitals. Official document to which the personnel involved in the intervention have access. Preventive measures will be focused on organizational, educational and behavioural elements related to older people and healthcare professionals:

- Support and instruction to the patient in preventive health measures (wear suitable footwear and, when necessary, include orthoses; repeated reminders to patients of the need to notify the team for any situation (getting up/lying in bed, going to the bathroom/shower, walking in the hallways, …)).
- Take extreme measures to prevent falls by healthcare professionals at times related to meals and transfers.
- Placing a poster encouraging the need to review medication(s) at admission and periodically through patient monitoring.
- Patient risk management procedures, including post-fall monitoring, alternatives to restrictions and/or restrictive elements, increased number of nursing visits to the patient, promotion of safe mobility and bathing practices based on specific clinical guidelines (Register Nurses’ Association of Ontario, 2011) and use of proper closed footwear.
- Monitoring and improvement in the distribution of work shifts in healthcare professionals.

### Data collection: Instruments

***Phases 1 and 2***: Several instruments will be used for data collection. The variables related to the fall and older person will be collected through the fulfilment of a specific record sheet for falls. This record sheet will have to be completed by the patient’s referring nurse (professional who generally records the fall, a fact documented in previous investigations [6]. The registration sheet will be the one attached at the end of this report, similar to that used in a previous project of the research group [20] (ANNEX I). To facilitate this process, an electronic data collection notebook will be hosted on the website of the study hospitals.

On the other hand, the sleep pattern will be measured by means of the Pittsburgh Sleep Quality Index (Spanish version) [29]. The chronotype of each person will be determined using the Horne-Östberg Morningness-Eveningness Questionnaire (Spanish version) [30]. For the quality of life related to health, the 36-Item Short Form Health Survey, version 2 (SF-36v2) (Spanish version) will be used [31], while for the general health status, the 28-Item General Health Questionnaire (GHQ28) (Spanish version) will be used [32]. The variables somnolence, fatigue and speed of response to the event will be measured by means of the Epworth Sleepiness Scale (Spanish version) [33].

With the intention of managing the data appropriately, an electronic data collection notebook will be developed by the Bioinformatics Unit of the Maimonides Institute for Biomedical Research of Córdoba.

***Phase 3***: The focus groups will be constituted with healthcare professionals—doctors, nurses and nursing assistants who provide and have provided their professional services in the field of study in the period previously indicated.

### Data collection: Procedure

***Phase 1***: The project will be explained to the institutional managers of the centres included in the study, who will appoint a coordinator to ensure proper development and will work in coordination with the research team. In these meetings, the process of recording falls will be disclosed, as well as the other variables.

Prior to data collection, meetings will be held with those responsible for the Clinical Management Units and special mention will be made of the areas that are expected to have a greater number of events (internal medicine, neurology, trauma, rehabilitation, …). Additionally, the dynamics of data collection will be explained.

On the other hand, being focused more on healthcare professionals (especially nurses) from the different study centres, several seminars will be held (first intervention), explaining how data collection should be done, as well as the definition of variables included in the record sheet. Such seminars will be proportional to the number of workers in the different centres included, being distributed over different days so that the number of workers receiving information is as large as possible. In addition, specific audits will also be carried out in each Clinical Management Unit.

During the first 18 months of study development, each time a fall occurs, in addition to recording the circumstances of the fall, it is necessary to obtain the informed consent of the patient to know their quality of life, general functional status, sleep pattern and chronotype. In healthcare professionals, informed consent must also be obtained to know the previous variables, as well as the values of fatigue/sleepiness, attention and cognitive functionality. In this sense, as it has been commented on in the inclusion/exclusion criteria, these variables will only be known in the healthcare professionals responsible for the older person, who have visualized and/or attended the fall event, even though the fall may be the result of a collective lack of attention from all the staff and not from a single person.

Once the prospective data collection period has started (January 2018), monthly individualized audits will be carried out in the different Clinical Management Units, aimed at managing correct data collection. Furthermore, these audits will allow obtaining a perspective of the fall event in relation to the number and related circumstances.

After obtaining and analysing the falls that occurred during the first 18 months of the study, time-adjusted preventive measures will be carried out. These measures will be focused on organizational, educational and behavioural elements (previously mentioned).

The establishment of the programme of time-adjusted prevention measures will be carried out in May 2019 in the four centres. The post-intervention data collection will cover July 2019 to September 2020. The managers responsible for each centre, together with the research team, will implement the measures, and, as in the first phase, seminars will be held with healthcare professionals to focus on the recording of falls and their circumstances, as well as the variables included in the prevention programme.

During the post-intervention phase, the individual monthly audits will continue to be carried out with each Clinical Management Unit. This follow-up will make it possible to discern the effectiveness of the preventive measures.

***Phase 2***: For the retrospective study, the data will be delivered by the centres participating in the project to the Principal Investigator anonymized.

***Phase 3***: The development of the focus groups will be carried out in the facilities of the participating hospitals, ensuring the confidentiality of the participants. Four focus groups will be held at the Reina Sofia University Hospital in Córdoba and one in each of the three regional centres.

### Data analysis

***Phases 1 and 2.*** For the analysis of quantitative data, descriptive statistics will be used, including measures of frequency, central tendency and dispersion according to the type of variable (quantitative or qualitative). Normality will be calculated with Shapiro-Wilk’s test and homogeneity with Levene’s test. To compare proportions of categorical variables, the chi-square test for contingency tables will be used. In the case of 2 × 2 tables, such tests with Yates correction will be used, and if the expected frequency is ≤ 5, Fisher’s exact test will be used. To compare mean values between the quantitative variables of two groups, Student’s t test for independent samples will be used. For the comparison of the mean values between quantitative variables and hospitals, the one-factor ANOVA test and ad-hoc tests with correction by GT2 Hochberg (if variance is homogeneous) or Games Howell (if variance is unequal) will be used. For the correlation between the quantitative variables, Pearson’s correlation coefficient will be used. For the logistic regression, multivariate analysis will be used.

All hypothesis tests will be bilateral. All tests with a confidence level of 95 % (p < 0.05) will be considered statistically significant.

Simple and multi-component Cosinor analyses will be used to explore temporal patterns [34]. This analysis uses the least squares method to approximate the time series of data to obtain one or more harmonics that fit the wavy shape that best explain the variance of the data set. The proportion of the overall variance (PR) represented by each harmonic component and the final multi-component curve function serve as goodness of fit parameters; the higher the PR is, the better representation by the total variance of the harmonic. The F-test statistic applied to the variance, represented by an approximation of the cosine curve versus a linear approximation of the time series data, was used to reject the null hypothesis of zero amplitude (absence of cyclical variation with respect to the period of time explored). The statistical significance of temporal patterns will be verified when the null hypothesis rejected for the F-test statistic is p < 0.5. The descriptive parameters of the statistically significant temporal patterns are the statistical estimated mean rhythm line (MESOR, the estimated rhythmic mean of the time series), amplitude (A, the numerical difference between the peak and valley values), temporal peak (φ, orthophase) and temporal valley (bathyphase); these last two variables are expressed according to the local midnight (00:00 h). The prominence of the temporal patterns, in terms of amplitude, will be expressed according to the MESOR as % AMP (greater prominence of the temporal variation, if the % AMP is greater). The variation will be low if % AMP < 25, moderate if 26–50, and high if > 50. To carry out the simple and multi-component Cosinor analyses, Chronolab software will be used [34].

***Phase 3.*** Analysis of the content of the focus groups will be carry out as follow: (i) transcription content; (ii) data reduction; (iii) exposure of the data; and (iv) conclusions and verification. The analysis of transcription content is approached from two perspectives—Analysis of the narrative (how is it counted?) and thematic/categorical analysis (what is counted?). In the data reduction phase, the facilitating method is the Giorgi Method (1997). The following steps are identified in it: reading the documents to obtain a global meaning; second reading to select the “units of meaning”, or fragments of the text that reveal something important regarding the object of the study; interpretation of meaning units to group them into “meaning statements”; statements of meaning are grouped into “common themes”; the relationship between meaning statements and common themes with the starting categories of the study;, and showing the emerging categories. This analysis will be complemented with the study of the annotations collected in the field notebook during the group interview. To conclude the analysis, a triangulation process will be carried out for researchers, study subjects and data collection techniques.

### Validity and reliability/Rigour

Multiple triangulation will be carried out with three types of data—strategies (quantitative and qualitative phases), techniques (longitudinal prospective study, documentary study and focus groups) and researchers. With the use of this methodology, the aim is to give the results obtained greater relevance and reliability [35].

## Discussion

In a multi-causal adverse event such as a fall, a good initial evaluation of hospitalized elderly people and their registration is necessary. The results of the study, in addition to stimulating the epidemiological investigation of falls, are intended to prevent falls in the hospital setting. Obtaining temporal patterns in hospital falls allows establishing high-risk periods during the day. This fact may correspond to certain activities predictable in time. In this sense, previous studies applied in Italian hospitals have shown that the establishment of time-adjusted preventive measures has led to a decrease in the number of falls [20, 21]. All this will contribute to improvement in the safety of the individual, and as consequence of their health, environment (family environment) and social level (due to the decrease in costs that a decrease in dependency implies) [20].

Furthermore, the study interventions emphasize the importance of recording in-hospital falls and their circumstances, especially time. Therefore, it represents a change in health policy. In previous studies [6], a low incidence of falls has been detected in the hospitals of Andalusia, being less than 1 %, although 65 year olds are the population that suffers the most from these types of events, which is contradictory. Contradiction contrasted with other studies [24], where healthcare professionals showed that there is an underestimation of hospital falls. On the other hand, the previous studies that support the present project [20] found high incidences as a result of an appropriate registry of these events.

Nurses routinely need 24 h per day to care for patients, in many different settings and environments. The impact on physical and psychological well-being of shift work management and lengths of shifts have been largely studied [36, 37]. The findings emerging from these investigations are that anomalies of the circadian rhythm summed with the characteristics of shift workers may have a direct influence on the health of nurses [38]. The outcome to be analysed in the proposed study relate chronotype, sleep quality and health status of the nurse with the quality of clinical practice and ultimately patient safety.

The findings that are expected to be found will be transferable to the institution and, in addition, the expected decrease in the incidence of falls will mean a reduction in healthcare costs. The findings endorsed by previous studies carried out in Italian hospitals [20, 39] showed interesting results.

The hourly categorization of fall events according to the evaluated intrinsic and extrinsic risk factors allows for a more precise understanding of their epidemiology and, ultimately, the improvement of the instituted prevention programme. Therefore, the results are likely to generate clinical practice guidelines [6, 24].

The results of this project are intended to promote a more exhaustive investigation of falls, thereby improving initiatives for their prevention. Furthermore, the study interventions emphasize the importance of recording in-hospital falls and their circumstances, especially time. The proposed registration system could be easily implemented and represents a change in health policy. The application of preventive measures according to time (*chronoprevention)* applied in this study will influence the reduction in the number of hospital falls considerably.

### Limitations

In the quantitative phases, the non-visualization of the fall may suppose an uncertain record of the event and its circumstances. Furthermore, both the record of falls and the section related to the state of health of the professionals are circumscribed to the idiosyncrasy of healthcare professionals. Limitations that are also present in the retrospective phase. A clinical trial could have more generalizability of the results and less bias. However, it should be noted that the interventions are established based on temporal patterns of incident events and relative risk factors for falls and related injuries in the participating hospitals. Therefore, with the intention of improving patient safety, the intervention is tailored to the casuistry of each centre.

The data obtained in the qualitative phase are subjective to perceptions of healthcare professionals. They cannot always express their opinions [40].

An information sheet on the study will be delivered to both the patient and the participating professionals, for which they will need to sign the informed consent.

For the retrospective study, the data will be delivered to the Principal Investigator anonymized.

## Supplementary Information

**Additional file 1:**

## Data Availability

The data that support the findings of this study are available from Andalusian Public Health System but restrictions apply to the availability of these data, which were used under license for the current study, and so are not publicly available. Data are however available from the authors upon reasonable request and with permission of Andalusian Public Health System.

## Abbreviations

AMP: Amplitude as % MESOR
GHQ28: 28-Item General Health Questionnaire
MESOR: The rhythm-estimated time series mean
PR: Proportion of overall variance
SF-36v2: 36-Item Short Form Health Survey, version 2
