# Supplementary material for: Chronoprevention in hospital falls of older people: protocol for a mixed-method study
Source: BMC Nurs. 2021 Jun 6;20:88. doi: 10.1186/s12912-021-00618-y (PMC8183051; doi:10.1186/s12912-021-00618-y)
Supplement: Supplementary file 1 — Additional file 1: [file 12912_2021_618_MOESM1_ESM.docx]

Annex I. Sheet registration of hospital falls

| **Hospital falls. Sheet registration** | | | |
| --- | --- | --- | --- |
| **Family Name (s):** | **Name:** | | **Sex:** [ ]M [ ]F |
| **Place of birth:** | **Date of birth:** | | **Date of fall:** |
| **Hospital:** | **Unit:** | | **Hour of fall:** |
| **Date of admission: __________________**  **Witness:** [ ] Patient [ ] Other patient [ ] Family Member [ ] Health professional [ ] Other ______________  **Situation:** [ ] Standing [ ] Sitting [ ] From bed, with bedrails [ ] From bed, no bedrails [ ] From armchair [ ] Other: _______________________________  **Cause:** [ ] Strength loss [ ] Balance loss [ ] Conscience loss [ ] Dizziness/Fainting [ ] Tumble [ ] Slip on dry surfaces [ ] Slip on wet surfaces [ ] Unknown [ ] Perceived [ ] Reported by [ ] Other__________________________________________  **Whereabouts:** [ ] Bathroom [ ] Room [ ] Corridor [ ] Public Area [ ] Stairs [ ]  Other__________________________________________  **Footwear:** Yes [ ] No [ ] Open: Yes [ ] No [ ]  **Nursing shift and number of workers on duty:**  _______________________________________________  _______________________________________________  **Nurse signature:**  _______________________________________________ | | **Nursing assesment** ____________________________________  ____________________________________  ____________________________________  **Reported injuries: _**___________________  ____________________________________  ____________________________________  ____________________________________  ____________________________________  **Examinations requiered:** [ ] TC [ ] X-Rays [ ] Other ____________________________________  ____________________________________  **Drugs administered (dose/time):** ___________________________________  ___________________________________  ___________________________________  ___________________________________  ___________________________________ | |
